# TLR2 Mediates Recognition of Live *Staphylococcus epidermidis* and Clearance of Bacteremia

**DOI:** 10.1371/journal.pone.0010111

**Published:** 2010-04-09

**Authors:** Tobias Strunk, Melanie R. Power Coombs, Andrew J. Currie, Peter Richmond, Douglas T. Golenbock, Liat Stoler-Barak, Leighanne C. Gallington, Michael Otto, David Burgner, Ofer Levy

**Affiliations:** 1 School of Paediatrics and Child Health, University of Western Australia, Perth, Australia; 2 Division Infectious Diseases, Children's Hospital Boston, Boston, Massachusetts, United States of America; 3 Harvard Medical School, Boston, Massachusetts, United States of America; 4 University of Massachusetts Medical School, Worcester, Massachusetts, United States of America; 5 National Institute of Allergy and Infectious Diseases, National Institutes of Health, Bethesda, Maryland, United States of America; 6 Murdoch Childrens Research Institute, Royal Childrens Hospital, Parkville, Victoria, Australia; Columbia University, United States of America

## Abstract

**Background:**

*Staphylococcus epidermidis* (SE) is a nosocomial pathogen that causes catheter-associated bacteremia in the immunocompromised, including those at the extremes of age, motivating study of host clearance mechanisms. SE-derived soluble components engage TLR2; but additional signaling pathways have also been implicated, and TLR2 can play complex, at times detrimental, roles in host defense against other *Staphylococcal spp*. The role of TLR2 in responses of primary blood leukocytes to live SE and in clearance of SE bacteremia, the most common clinical manifestation of SE infection, is unknown.

**Methodology/Principal Findings:**

We studied TLR2-mediated recognition of live clinical SE strain 1457 employing TLR2-transfected cells, neutralizing anti-TLR antibodies and TLR2-deficient mice. TLR2 mediated SE-induced cytokine production in human embryonic kidney cells, human whole blood and murine primary macrophages, in part via recognition of a soluble TLR2 agonist. After i.v. challenge with SE, early (1 h) cytokine/chemokine production and subsequent clearance of bacteremia (24–48 h) were markedly impaired in TLR2-deficient mice.

**Conclusions/Significance:**

TLR2 mediates recognition of live SE and clearance of SE bacteremia *in vivo*.

## Introduction

*Staphylococci spp.* are frequent causes of nosocomial infection [1], [2]. Coagulase-negative staphylococci, especially *S. epidermidis* (SE) are ubiquitous skin commensals and a major cause of nosocomial bacteremia, particularly in immunocompromised patients and those with implantable medical devices [3]. The ability of *Staphylococcus spp.* to cause disease has been ascribed to immune evasion, including avoidance of opsonophagocytosis [4]. SE lacks genome islands found in *S. aureus* (SA) encoding pathogenesis factors, likely contributing to its lesser virulence [5]. Nevertheless, SE is a frequent cause of bacteremia, especially at the extremes of age, resulting in significant morbidity and mortality [6]. SE attaches to foreign material, interacts with the host extracellular matrix and elaborates biofilm, reducing deposition of complement/IgG, susceptibility to host antimicrobial peptides and neutrophil-mediated killing [3], [7], [8].

Characterization of the *Toll*-like receptor (TLR) system [9], [10], [11], [12] has paved the way for studies implicating TLR2 in recognition of Gram-positive bacteria [9], [10], [13]. However, additional recognition pathways of Gram-positive bacteria include β-integrins [14], [15], lectins [16], CD36 [17], and nucleotide oligomerization domain proteins 1 and 2 (NOD1 and -2) [18], members of the NOD-like receptor (NLR) family of cytosolic sensors [19]. SA-derived factors activate NODs1 and -2 and the NLRP3 inflammasome [18], [20] and NOD2 contributes to cutaneous defense against live SA *in vivo* [21].

There is evidence both in favor and against a role for TLR2 in host defense against several Gram-positive bacteria, including SA [18], [22], [23], [24]. The complexity of the data may relate to distinct routes of infection in murine models- e.g., i.p. vs. i.v. [22], [25], differences in bacterial preparations (e.g., live vs. killed) and inoculum size, and differences in innate immunity between mice and humans [26]. Live SA subverts TLR2 to inhibit superoxide production by murine macrophages thereby prolonging survival of SA in phagosomes [23]. Thus the roles of TLR2 in innate responses to SA are context dependent, and may include detrimental roles in infection outcome.

SE also engages the innate immune system [6]. Killed SE preparations induce cytokine production from human peripheral blood mononuclear cells *in vitro* [27], [28], [29], [30], and fractions containing SE-derived phenol-soluble modulin peptides (PSMs) induce cytokine production in TLR2-transfected HEK cells and primary murine cells [31], [32], [33], [34]. SE surface polysaccharide intercellular adhesin activates human astrocytoma cells via TLR2 [35]. In contrast, SE-derived peptidoglycan (PG) activates human monocytic THP-1 cells via both TLR2 (polymeric PG) and NOD2 (monomeric PG) [36].

Although SE-derived factors can activate human cultured cells via TLR2 and NOD2, their relative contribution to responses of primary leukocytes to live SE *in vitro* or clearance of SE bacteremia *in vivo*- experimental settings that may most closely mimic clinical infection- are undefined. As bacteremia is the major clinical manifestation of SE infection, characterizing bloodstream clearance mechanisms is a priority. We therefore investigated the interaction of live SE with TLR2, studying both human and murine cells *in vitro* and intravenous infection of mice *in vivo*, thereby avoiding potential limitations inherent to study of killed bacteria, isolated bacteria-derived factors, or analyses relying on a single mammalian species [26], [37]. We demonstrate for the first time that live SE, which elaborates a TLR2-activating soluble factor (SE-S), can activate primary cells via human and murine TLR2 *in vitro* and that TLR2 substantially and selectively contributes to clearance of SE bacteremia *in vivo*.

## Methods

### Ethics statement

Blood was collected from healthy donors after written informed consent in accordance with the institutional review board-approved study protocols of Children's hospital Boston (X07-05-0223). All animal protocols were approved by the Animal Care and Use Committee of Children's Hospital Boston (08-11-1261R).

### Bacteria

Wild-type (WT) SE strain 1457, obtained from a patient with an infected central venous catheter [38], was grown to mid-log phase in Brain Heart Infusion Broth (PathWest, Perth, Australia), collected by centrifugation and resuspended in pyrogen-free PBS. Lipopolysaccharide (LPS) contamination of SE preparations was excluded by *Limulus* amebocyte lysate assay (Associates of Cape Cod, East Falmouth, MA) and lack of response by TLR4-transfected HEK293 cells (Invivogen, San Diego, CA). For study of the SE-derived soluble factor (SE-S), SE were grown overnight in trypticase soy broth (TSB), centrifuged at 2900×g at 4°C for 5 min and the supernatant filtered (0.2 µm) and stored at −20°C until use.

### TLR agonists

TLR agonists used in this study included fibroblast-stimulating lipopeptide-1 (FSL-1, TLR2/6, 1 µg/ml; InvivoGen), ultra-pure LPS from *Salmonella minnesota* devoid of TLR2-stimulating activity (TLR4; 100 ng/ml; List Biological Laboratories, Campbell, CA).

### Whole Blood

Blood was collected either in sodium heparin tubes (Vacutainer; Becton Dickinson or Greiner, Kremsmünster, Austria) and tested whole, or mixed 1∶1 with RPMI 1640 (Gibco, Life Technology, Paisley, Scotland) prior to culture in polypropylene tubes or in round-bottom 96-well plates (Corning Incorporated) at 37°C/5% CO_2_. Culture supernatants were harvested and stored at −20°C.

### Antibody blocking

Heparinized whole blood was incubated with 10 µg/ml neutralizing, azide- and antibiotic-free, IgA mAbs to human TLR2 or TLR4 (Invivogen) for 30 min prior to addition of FSL-1 (1 µg/ml), LPS (100 ng/ml) or SE (10^6^/ml). After 4 h, culture supernatants were harvested and stored at −20°C prior to IL-6 ELISA. To exclude effects on bacterial viability, blood was plated onto blood agar plates and CFUs enumerated, after overnight incubation (37°C/5% CO_2_). For blocking SE-S, whole blood was incubated with neutralizing azide-free rat polyclonal Abs against human TLR2, TLR4, TLR6 or isotype control (Invivogen) for 30 min prior to addition of stimulus. After 18 h, culture supernatants were harvested and stored at −20°C.

### TLR-transfected human embryonic kidney cells

Stable cell lines of human embryonic kidney (HEK)-293 cells transfected with TLR2 or TLR4/MD2 were cultured in supplemented DMEM containing 10% heat-inactivated FCS and 10 µg/ml ciprofloxacin [39]. After antibiotic removal, cells were stimulated with SE (10^6^–10^8^ bacteria/ml) or pure TLR agonists for 24 h (5%CO_2_, 37°C), and cell culture supernatants harvested for storage at −20°C.

### Cytokine Assays

Human cytokines were measured by time resolved fluorometry (TRF; IL-6), commercial ELISA (IL-8 and IL-6; eBioscience, San Diego, CA) per manufacturers' instructions, using Maxisorp flat-bottom plates (Nunc, Roskilde, Denmark) [40] or by employing multi-analyte flurometric beads (Milliplex, Millipore) on a Luminex xMAP system (Luminex Corp., Austin, TX) using BeadView Multiplex Software v.1 (Upstate Cell Signaling Solutions, Temecula, CA).

### Mice

TLR2-deficient (Jackson laboratories; Bar Harbor, ME) and C57BL/6 WT (Taconic; Hudson, NY) female mice were confirmed by genotype (PCR) and phenotype (impaired peritoneal macrophage response to FSL-1 with preserved response to LPS) and matched for age.

### Bacteremia Model

Adult mice 7–12 weeks old were injected i.v. with 10^8^ SE. Blood was collected by submandibular bleed at 1, 4, 24 and 48 h post-infection. For cytokine analysis, blood was diluted (5 volumes RPMI) and plasma collected (1200×g at 4°C; 5 min) and stored at −20°C.

### Primary peritoneal macrophages

Murine macrophages were obtained from the peritoneal cavity of mice 3 days after i.p. thioglycollate injection. Adherent cells were maintained in DMEM supplemented with 10% FCS and ciprofloxacin (10 µg/ml) at 5% CO_2_/37°C. Following ciprofloxacin removal by washing with PBS, cells were stimulated with SE (10^4^–10^8^/ml) for 4 h and cell culture supernatants stored at −20°C.

### Trans-well assays

SE were added to the upper chamber of a 0.4 µm trans-well system in a 24 well plate (Corning Costar; Acton, MA) in DMEM with 10% FCS for 4 h. There were no live SE found in lower chamber. Cell culture supernatants were harvested and stored at −20°C prior to cytokine measurement.

### Murine cytokines

Murine cytokines were determined by ELISA (TNF and IL-6; R&D Systems; Minneapolis, MN) or by employing multi-analyte flurometric beads (Milliplex, Millipore) on a Luminex xMAP system (Luminex Corp., Austin, TX) using BeadView Multiplex Software v.1 (Upstate Cell Signaling Solutions, Temecula, CA).

### Statistical Analyses

Statistical analyses employed Graphpad Prism v5.0 for Macintosh (GraphPad, La Jolla, CA). Unless stated otherwise in the figure legend, comparisons between 2 groups were by the Mann-Whitney U test and those between >2 groups were by the Kruskal-Wallis test with Dunn's post-test. P-values <0.05 were considered statistically significant.

## Results

### Transfection of TLR2 into HEK cells confers responsiveness to SE

**Figure 1:**
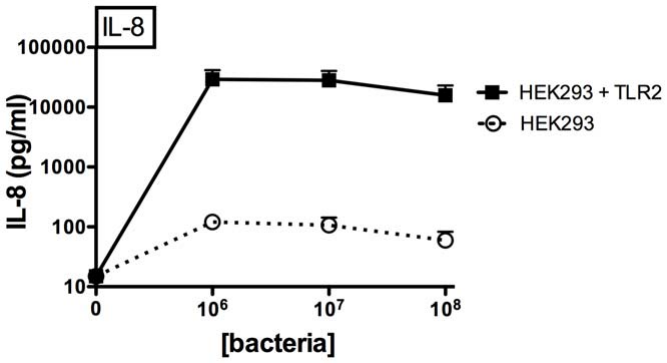
Transfection of human embryonic kidney cells with TLR2 confers responsiveness to live SE. Transfection of HEK293 cells with TLR2 enhanced SE-induced IL-8 production after 24 h of stimulation (n = 3).

We first confirmed that live SE could engage TLR2 to induce cytokine production by assessing TLR2-transfected HEK cells. TLR2-transfected HEK cells demonstrated dramatically increased IL-8 production in response to SE (Fig. 1), indicating that live SE engages TLR2. In contrast, transfection of HEK cells with TLR4/MD-2 did not enhance SE-induced IL-8 production (data not shown).

### SE-induced cytokine production is inhibited by blocking TLR2 in whole blood

**Figure 2:**
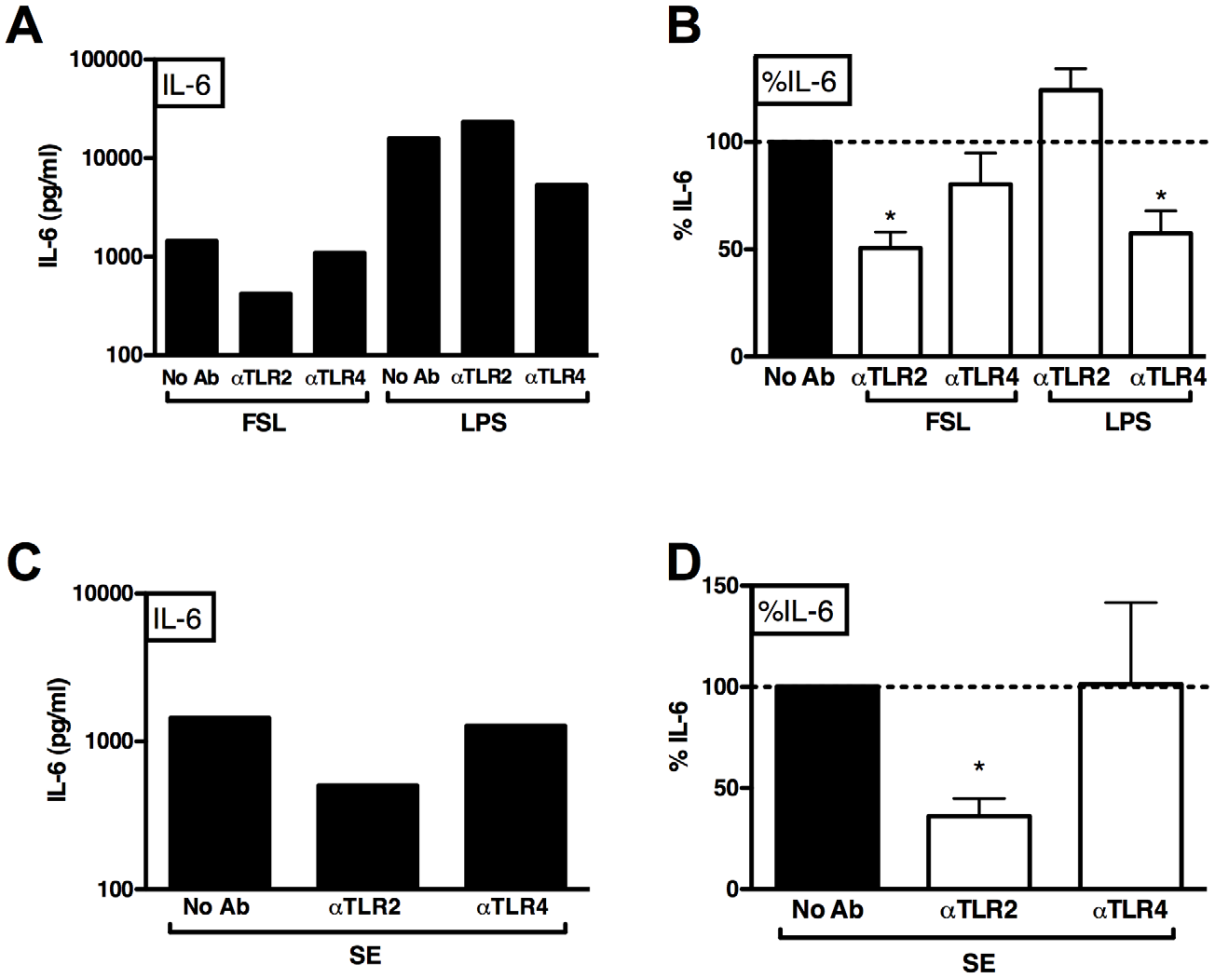
Neutralizing anti-TLR2 antibody inhibit SE-induced cytokine production in human blood. Blocking anti-TLR IgA Abs were added to human whole blood (10 µg/ml) 30 min prior to stimulation with SE (10^6^/ml) for 4 h and extracellular IL-6 measured by ELISA. (A) Anti-TLR2 Ab selectively inhibited FSL-, but not LPS-induced IL-6 production (representative of four similar experiments). (B) Composite analysis of inhibition expressed as %IL-6 production relative to control (no Ab); n = 4; (C) Selective inhibition of SE-induced IL-6 by anti-TLR2, but not anti-TLR4 Ab (representative of 4 similar experiments). (D) Composite analysis of inhibition expressed as %IL-6 production, relative to control (no Ab). 1 sample-t-test vs. 100% (n = 4); * p<0.05.

To assess the potential role of TLR2 in SE-induced innate immune responses in human blood, we investigated the effect of blocking anti-TLR Abs on SE-induced IL-6 production in human whole blood (Fig. 2). Pre-incubation of human whole blood with neutralizing anti-TLR2 and TLR4 Abs (10 µg/ml) inhibited cytokine responses to FSL-1 and LPS, respectively (Fig 2A & B). Pre-incubation of human whole blood with anti-TLR2 Ab inhibited SE-induced IL-6 production by >50%, whereas pre-incubation with anti-TLR4 had no effect (Fig. 2C & D).

### Impaired SE-induced TNF production in TLR2−/− murine peritoneal macrophages

**Figure 3:**
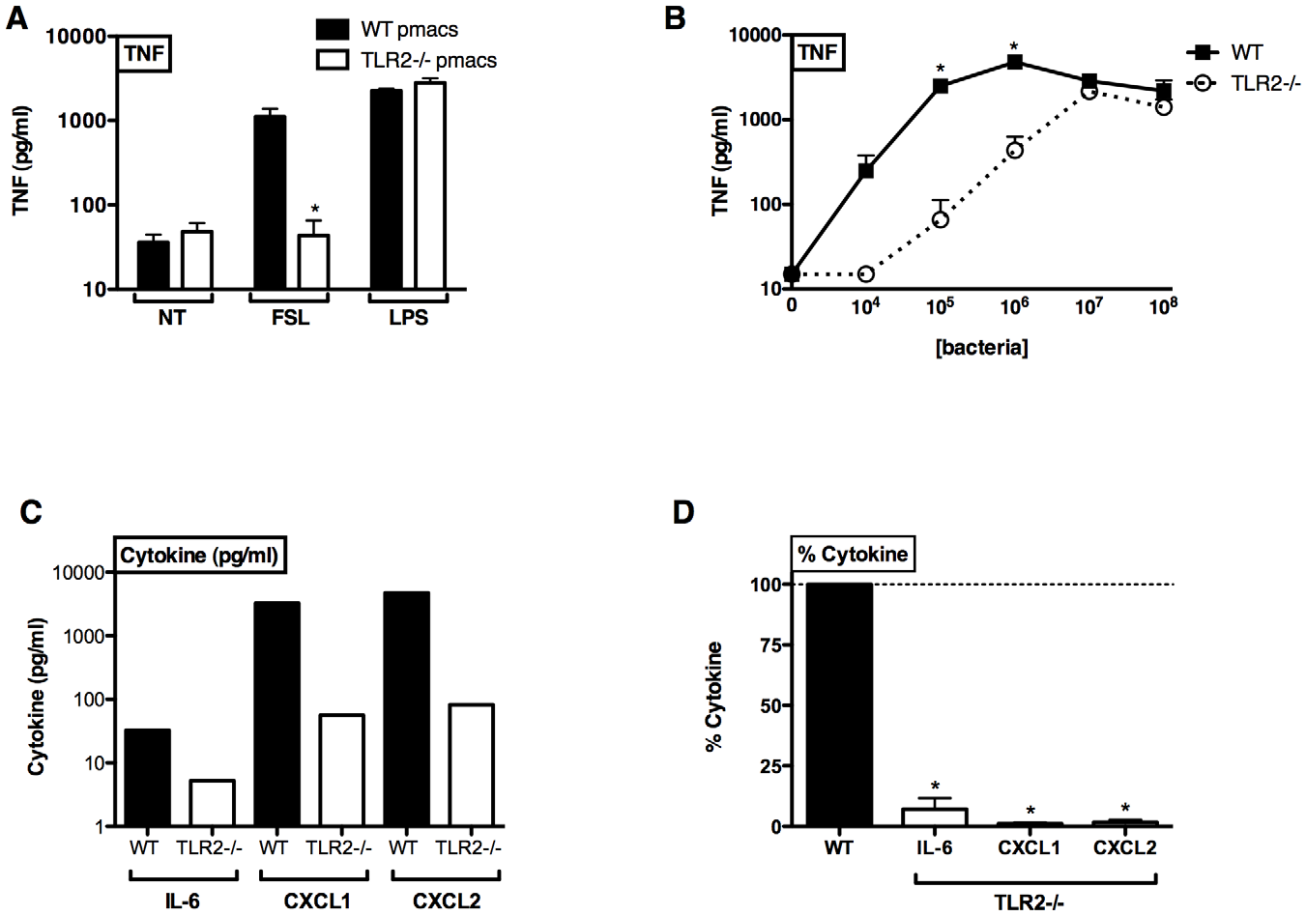
Impaired SE-induced cytokine production from primary TLR2−/− murine peritoneal macrophages. Primary murine peritoneal macrophages (pmacs) from WT and TLR2-deficient C57BL/6 mice were stimulated with SE for 4 h. (A) Selectively impaired bacterial lipopeptide FSL-1-induced cytokine response in TLR2-deficient pmacs, with normal responses to LPS (TLR4). (B) TLR2-deficient pmacs demonstrated markedly impaired TNF production to low concentrations (10^4^–10^6^ bacteria/ml) of SE (n = 3–9). * p<0.05. (C) SE-induced production of IL-6, CXCL1, and CXCL2 was lower in TLR2-deficient pmacs (representative of 3 similar experiments). (D) Composite analysis demonstrating % cytokine production of TLR2-deficient pmacs relative to WT (n = 3, one-sample t-test versus 100%). *p<0.05.

We subsequently measured cytokine responses to SE in peritoneal macrophages derived from wild-type and TLR2-deficient mice. As expected, TLR2-deficient peritoneal macrophages demonstrated marked impairment in TNF production in response to FSL-1 (TLR2/6) but equivalent response to LPS (TLR4) (Fig. 3A). TLR2-deficient macrophages demonstrated marked impairment in SE-induced TNF, particularly at lower concentrations of SE tested (10^4^–10^6^ CFU/ml; Fig. 3B). In contrast, TNF responses to high concentrations of SE (10^7^–10^8^) were largely TLR2-independent (Fig. 3B). Four hours after infection with 10^5^ LSE, TLR2-deficient macrophages also demonstrated diminished production of IL-6, as well as the chemokines CXCL1 and CXCL2 (Fig. 3C & D).

### SE secretes a soluble TLR2 agonist

**Figure 4:**
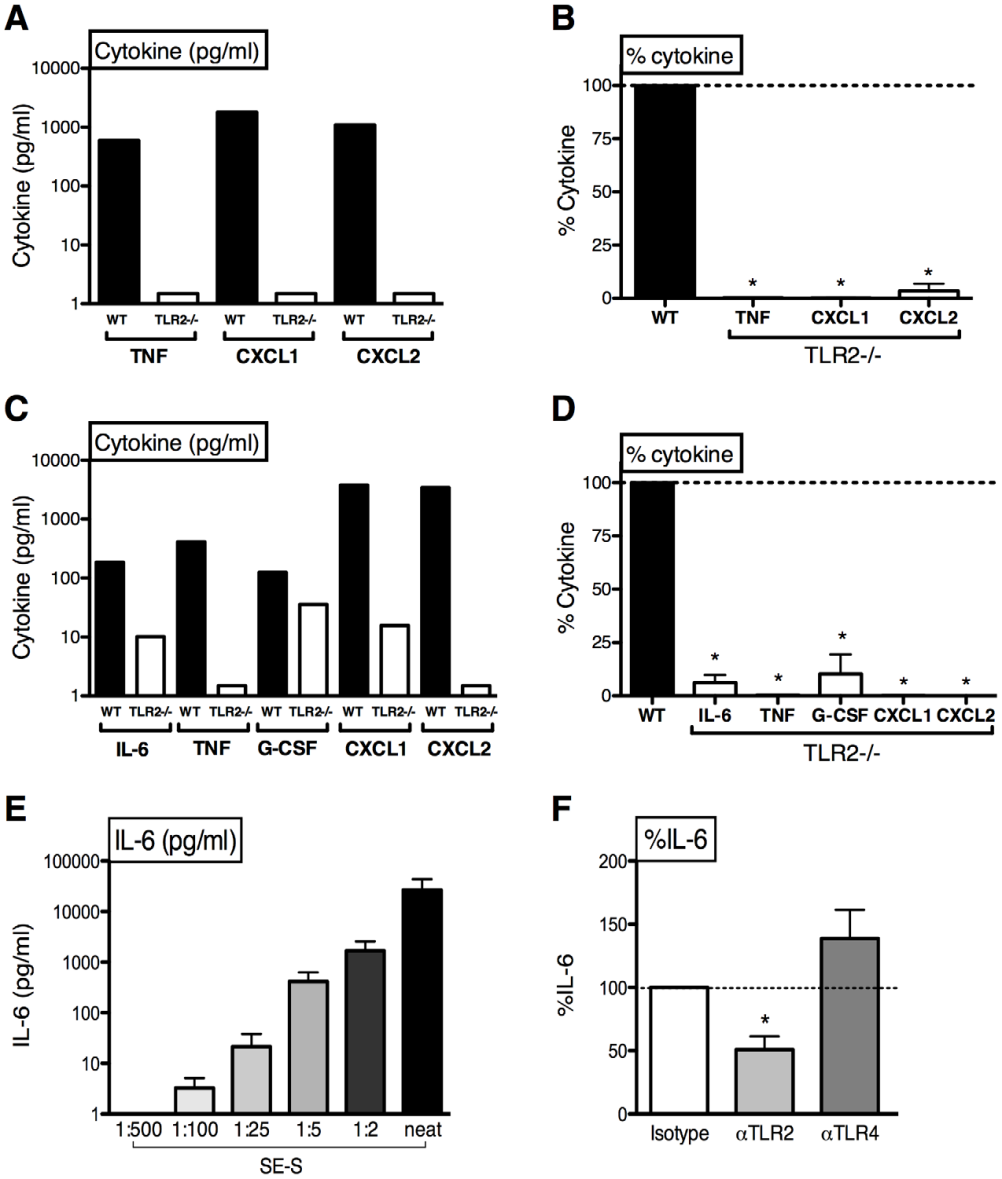
A soluble factor from SE activates cytokine production in a TLR2-dependent manner. (A) SE in a trans-well upper chamber induced cytokine production in WT, but not TLR2-deficient murine peritoneal macrophages (pmacs; representative of 3 similar experiments); (B) Composite trans-well analysis expressed as %IL-6 production in TLR2-deficient vs. WT pmacs (n = 3, 1-sample t-test vs. 100%); (C) Filtered supernatants of stationary phase SE contain a soluble factor (SE-S) which induced diminished cytokine/chemokine production from TLR2-deficient pmacs (representative of 3 similar experiments); (D) composite analysis of SE-S-induced cytokine/chemokine production in TLR2-deficient vs. WT pmacs (n = 3, one-sample t-test versus 100%). (E) SE-S induced concentration-dependent IL-6 production in human whole blood, (F) neutralizing anti-TLR2 polyclonal Abs, but not anti-TLR4 polyclonal Abs, blunted SE-S-induced IL-6 in human whole blood. * p<0.05.

We next assessed whether the TLR2 agonist activity of SE may be released as a soluble factor. In trans-well experiments in which SE were placed in the upper chamber, wild-type murine macrophages in the lower chamber produced TNF, CXCL1 and CXCL2 suggesting the secretion of a soluble factor that could traverse the semi-permeable membrane (Fig. 4A). This SE-derived secreted factor (SE-S) was highly TLR2-dependent as responses of TLR2-deficient macrophages were dramatically impaired (Fig. 4A, and composite analysis in Fig. 4B). Accordingly, TLR2-deficient pmacs demonstrated impaired SE-S-induced cytokine and chemokine production (Fig. 4C & D). SE-S also induced IL-6 production in human whole blood in a concentration-dependent manner (Fig. 4E), an activity that was inhibited by neutralizing anti-TLR2 Abs (Fig. 4F).

### SE-induced cytokine production *in vivo* is mediated via TLR2

**Figure 5:**
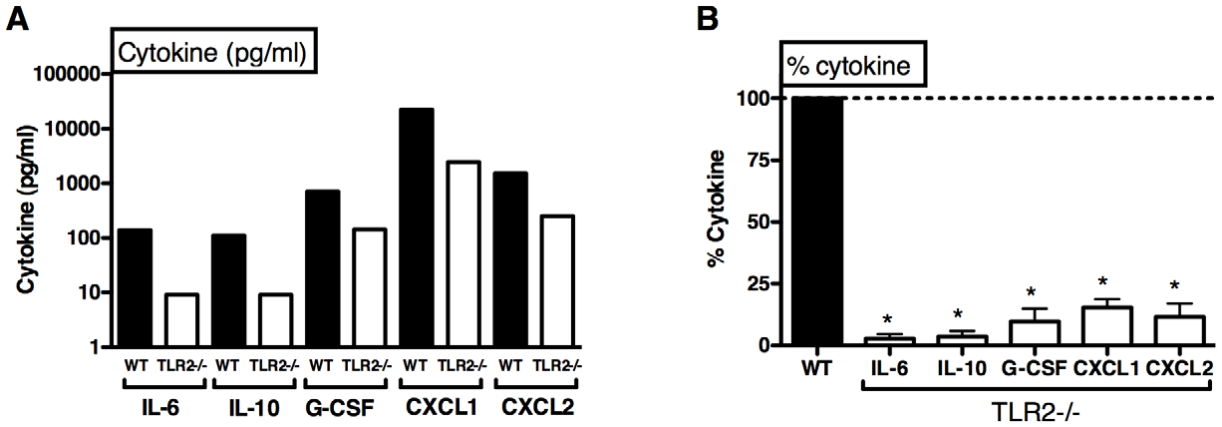
SE-induced cytokine production is TLR2-dependent *in vivo*. Female C57BL/6 WT or TLR2-deficient mice were injected intravenously with 10^8^ CFU SE prior to collection of peripheral blood at 1 h for measurement of cytokines/chemokines by using multi-analyte flurometric beads as described in Methods. (A) SE-induced production of cytokines/chemokines is lower in TLR2-deficient mice (representative of 3 similar experiments). (B) Composite analysis demonstrating % cytokine production of TLR2-deficient mice relative to WT (n = 3, one-sample t-test versus 100%). *p<0.05.

To test whether TLR2 contributes to SE-induced cytokine production in peripheral blood *in vivo*, we injected SE i.v. to wild-type or TLR2-deficient mice then measured cytokine production by collecting blood plasma at 1 h (Fig. 5). TLR2-deficient mice demonstrated markedly diminished production of IL-6, IL-10, G-CSF, CXCL1 and CXCL2 *in vivo* (Fig. 5A), that was significant upon composite analysis (Fig. 5B).

### TLR2 mediates clearance of SE bacteremia *in vivo*

**Figure 6:**
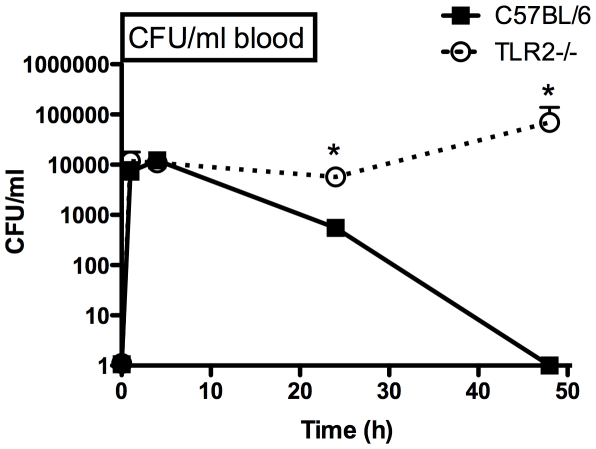
TLR2-deficient mice demonstrate impaired clearance of SE bacteremia. Female C57BL/6 WT or TLR2-deficient mice were injected i.v. with 10^8^ CFU of SE prior to collection of peripheral blood at 1, 4, 24 or 48 h for measurement of CFUs by plating serial dilutions (n = 4–9); *p<0.05.

To assess the role of TLR2 in clearance of SE, C57BL/6 wild-type or TLR2-deficient mice were injected i.v. with SE (10^8^ CFU) prior to collection of blood for analysis. TLR2-deficient mice demonstrated impaired clearance of SE bacteremia, with dramatically and significantly higher peripheral blood concentrations of SE at 24 and 48 h (Fig. 6).

## Discussion

We have demonstrated for the first time the importance of TLR2 in recognition of live SE as measured by whole blood cytokine production and highlight a selective role of TLR2 in clearance of SE bacteremia. TLR2 mediated SE-induced cytokine production in TLR2-transfected HEK cells, human whole blood, and murine primary peritoneal macrophages. TLR2-deficient mice injected intravenously with SE demonstrated reduced early (1 h) cytokine/chemokine induction and selectively and markedly impaired subsequent clearance of bacteremia at 24–48 h.

SE releases an as yet unidentified soluble factor (SE-S) that activates both human and murine cells via TLR2 (Fig. 4C & D). We speculate that release of SE-S may contribute to responses to SE *in vivo*. Indeed, our evidence that TLR2 plays a prominent role in recognition of, and responses to, SE is consistent with the ability of SE-derived surface components to induce inflammation [41], including TLR2 agonists such as PSM [31], [33], surface polysaccharide intercellular adhesin [35] and peptidoglycan [36]. Whether the SE-S activity detected in our present study reflects these or other molecules [42] will be the subject of future work.

Impaired production of cytokines in response to intravenous SE was associated with impaired clearance of bacteremia, suggesting that TLR2-mediated cytokine production may contribute to clearance of SE *in vivo*. In contrast, Raby et al recently reported that after i.p. challenge with SE, administration of inhibitory soluble TLR2 (sTLR2) reduced peritoneal PMN infiltration, but did not reduce bacterial clearance from the peritoneal cavity [32]. Our distinct results likely reflect different routes of bacterial challenge and different approaches to TLR2 inhibition, with genetic ablation providing a more definitive approach. SE engages TLR2, but at high bacterial concentrations can activate primary murine macrophages via a TLR2-independent pathway, indicating engagement of additional pattern recognition pathways, possibly including NLRs and the inflammasome [36], [37], [43].

Multiple aspects of our studies of live SE are novel: a) SE (and SE-S) activates cytokine production via TLR2 in human whole blood, b) TLR2 is particularly important in detecting low concentrations of SE in primary murine macrophages, whereas high concentrations of SE can activate these cells in a TLR2-independent manner, c) TLR2 mediates early (1 h) cytokine production after i.v. challenge with SE *in vivo* and d) TLR2 plays a crucial and selective role in mediating subsequent clearance of SE bacteremia at 24–48 h *in vivo*. We speculate that in TLR2-deficient animals, reduced production of early (1 h) plasma cytokines, including IL-6, impairs subsequent clearance of staphylococci, as has also been suggested in humans with neutralizing anti-IL-6 auto-antibodies [44].

In conclusion, our study is the first to demonstrate the importance of TLR2 in recognition of live SE in whole blood, a key site of SE infection, *in vitro* and *in vivo*. We have demonstrated a selective role of TLR2 in clearance of SE bacteremia, the most common, harmful and costly clinical manifestation of SE infection [3], [4]. Our study may inform efforts to develop novel adjunctive approaches to prevent and/or treat SE infection [45]. Novel TLR2 antagonists in biopharmaceutical development may reduce SE-induced inflammation, but may also impair clearance of SE bacteremia. Conversely, enhancing TLR2-mediated host defense may hasten clearance of SE bacteremia; consistent with protection against bacteremia afforded by hypermorphic alleles of TIRAP, a signaling molecule downstream of TLR2 [46]. Future studies should define the roles of TLR2 in susceptibility of immunocompromised populations, particularly those at the extremes of age, to SE bacteremia [6].
